# Effects of CO_2_-induced pH reduction on the exoskeleton structure and biophotonic properties of the shrimp *Lysmata californica*

**DOI:** 10.1038/srep10608

**Published:** 2015-06-01

**Authors:** Jennifer R. A. Taylor, Jasmine M. Gilleard, Michael C. Allen, Dimitri D. Deheyn

**Affiliations:** 1 Scripps Institution of Oceanography, Marine Biology Research Division, University of California, San Diego; 2 Division of Biological Sciences, University of Montana

## Abstract

The anticipated effects of CO_2_-induced ocean acidification on marine calcifiers are generally negative, and include dissolution of calcified elements and reduced calcification rates. Such negative effects are not typical of crustaceans for which comparatively little ocean acidification research has been conducted. Crustaceans, however, depend on their calcified exoskeleton for many critical functions. Here, we conducted a short-term study on a common caridean shrimp, *Lysmata californica*, to determine the effect of CO_2_-driven reduction in seawater pH on exoskeleton growth, structure, and mineralization and animal cryptic coloration. Shrimp exposed to ambient (7.99 ± 0.04) and reduced pH (7.53 ± 0.06) for 21 days showed no differences in exoskeleton growth (percent increase in carapace length), but the calcium weight percent of their cuticle increased significantly in reduced pH conditions, resulting in a greater Ca:Mg ratio. Cuticle thickness did not change, indicating an increase in the mineral to matrix ratio, which may have mechanical consequences for exoskeleton function. Furthermore, there was a 5-fold decrease in animal transparency, but no change in overall shrimp coloration (red). These results suggest that even short-term exposure to CO_2_-induced pH reduction can significantly affect exoskeleton mineralization and shrimp biophotonics, with potential impacts on crypsis, physical defense, and predator avoidance.

Great progress has been made toward understanding the full ramifications that ocean acidification will have on marine life, and the overall consensus is that the potential impact is generally negative, especially in combination with other stressors^1^. It is also clear that the specific effects of ocean acidification are complex and highly variable across the realm of biological complexity, depending on local environmental stressors and the ecophysiology of species, their biology, and life history^1^,^2^,^3^. Most of our current knowledge on invertebrates stems from studies that have focused primarily on molluscs, echinoderms, and cnidarians. The special scrutiny given to these marine organisms is because their calcification process can be greatly disturbed by changing ocean carbon chemistry, making them vulnerable to ocean acidification^4^,^5^,^6^. Indeed, several representative species from these groups of marine calcifiers tend to exhibit mostly negative effects of different physiological responses to OA, including, for example, dissolution of the calcified skeleton and reduced calcification rates^7^, metabolic depression^8^, reduced growth rate^9^,^10^, and reduced energy storage ability^11^.

Noticeably lagging in this growing body of knowledge is the response of crustaceans to ocean acidification. This is surprising given their commercial importance and characteristic calcified exoskeleton, which could make them vulnerable to changes in ocean pH. Furthermore, crustaceans may respond to increased acidity relatively quickly because they repeatedly shed their exoskeleton (cuticle) and secrete a new one in the process of moulting. One reason for the lack of focus on crustaceans may be attributed to their acid-base regulation ability, which helps buffer disruptions to internal pH, thereby making the calcification process less susceptible to changes in water chemistry in relation to pCO_2_ and carbonate balance^12^,^13^,^14^,^15^. Another potential reason is that, like in molluscs, the calcification process may not be dependent on environmental CO_3_^−^ saturation levels, but rather on bicarbonate (HCO_3_^−^) and metabolic CO_2_ instead^16^,^17^. Furthermore, the use of calcite, a less soluble form of CaCO_3_, in the exoskeleton may confer the ability to tolerate a larger variation of ocean carbonate chemistry, and provide more resilience (less sensitivity) to decreasing levels of carbonate saturation thresholds in seawater^16^,^17^. Indeed, no net changes in calcification after exposure to ocean acidification conditions were observed, for example, in the barnacle, *Elminius modestus*^18^, or the crab, *Necora puber*^14^.

Although the impact of OA on net calcification appears neutral, there is evidence that multiple crustacean species increase calcification in reduced pH conditions, which perhaps appears counterintuitive and an exception compared to what has been learned from most other marine calcifiers. Such crustaceans species include the barnacle, *Amphibalanus amphitrite*^19^, prawn, *Panaeus occidentalis*^20^, crab, *Callinectes sapidus*^21^, shrimp, *Penaeus plebejus*^21^, and lobster, *Homarus americanus*^21^, all of which showed increases in net calcification. The metabolic investment in increased calcification may, however, come at the expense of other physiological processes. Indeed, a series of negative effects from ocean acidification conditions, such as reduced growth and increased mortality, were observed in tanner and red king crabs^22^, barnacle larvae (*Amphibalanus improvises*)^23^, and the shrimp, *Palaemon pacificus*^24^. Reduced pH combined with increased temperature also causes metabolic depression in the porcelain crab, *Petrolisthes cinctipes*^25^.

Of the assorted crustacean species studied to date, most have shown either no net change or a net increase in calcification during exposure to low pH conditions^26^. One exception is that the second antennae of the shrimp *Palaemon pacificus* became severely shortened in low pH conditions, indicating that some species, and even specific calcified structures, can be vulnerable to dissolution^24^. While there appears to be consensus thus far for the impact on crustacean calcification, too little research has been conducted to be able to address the variability and complexity of species’ responses, especially to address the situation for species of economic importance. Clearly much more research is needed to gain a comprehensive understanding of how this large and diverse group of marine calcifiers responds to ocean acidification conditions. In particular, most researchers studying crustaceans measure calcification through changes in dry weight of the exoskeleton and spectrophotometry, but have not examined details of the calcification process, potential changes to the structure of the cuticle, nor the functional consequences of altered cuticle calcification.

The crustacean cuticle is a rather complex structure, despite it being shed and secreted repeatedly during growth. The cuticle is subdivided into four structurally distinct layers, from outermost to innermost: epicuticle, exocuticle, endocuticle, and membranous layer. The epicuticle is relatively thin and contains lipoproteins impregnated with calcium salts, but lacks chitin and lamellar organization^27^,^28^. Both the exocuticle and endocuticle layers are composed of chitin-protein fibers with mineral in between. These chitin-protein fibers are arranged in layers, with each layer occurring at a slight angle relative to the one above, forming a Bouligand structure^29^. The membranous layer contains no mineral and is composed of chitin and protein^30^. The primary mineral that impregnates the chitin-protein matrix is calcium carbonate, either in the form of crystalline calcite or amorphous calcium carbonate, and magnesium and phosphorous to a lesser extent^31^,^32^,^33^,^34^. Given the complexity of the crustacean cuticle, increased calcification could manifest itself in ways that could alter the mechanical integrity of the structure and its associated biological and ecological functions.

Changes to the structure and integrity of the crustacean cuticle could impact animals in multiple ways, as the exoskeleton supports many vital functions. Many caridean shrimp, for example, rely on transparency of the exoskeleton for camouflage from predators or use coloration for crypsis or recognition by potential mates and symbiotic species^35^,^36^. Transparency and coloration, which depends on the visibility of chromatophores through the cuticle, are both largely dependent on the ultrastructure of the exoskeleton, in particular its ability to minimize light scattering^37^. Changes in cuticle structure and calcium carbonate content due to ocean acidification could cause discontinuities in the refractive index of the shrimp body, ultimately reducing transparency and having negative consequences for their ability to avoid detection by predators.

While there is evidence that some crustaceans respond to reduced pH by increasing calcification, it remains to be determined how exposure to ocean acidification conditions affects the structure of the crustacean exoskeleton and how these potential structural changes affect higher level animal function and ecology. Here we conducted a short-term study on the effects of CO_2_-induced ocean acidification on the red rock shrimp, *Lysmata californica*, to decipher changes in exoskeleton ultrastructure and animal biophotonic properties.

## Materials and Methods

### Animals and experimental design

Twenty-four red rock shrimp, *Lysmata californica,* (see supplementary material, Fig. S1) were collected offshore San Diego, CA and brought to the Marine Biology Research Division experimental aquarium at Scripps Institution of Oceanography (SIO). Animals were blotted dry, weighed, and photographed using an HD digital camera (Leica DFC290, IL, USA) attached to a stereomicroscope (Leica M165 C, IL, USA). Carapace length was measured from the center of the left orbit, to avoid potential damage and variability of the rostrum, to the center of the posterior edge of the carapace using imaging software (LAS 4, Leica Microsystems, IL, USA), or digital calipers for the largest animals. Animals ranged in size from 2.4 to 20.8 mm carapace length and were divided so that the control and treatment had representatives spanning this size range.

The experimental set-up consisted of two tank systems that received filtered seawater pumped in from the SIO pier at ambient pH (7.99 ± 0.04), temperature (18.7 °C ± 0.9), and salinity (33-35 PSU). Each tank system included a large header tank (150 L) that fed flow-through seawater separately into 12 smaller experimental tanks (2.8L), each of which housed an individual shrimp. One tank system served as a control and was kept at ambient pH (7.99 ± 0.04, pCO_2_ = 462 ± 11 μatm; mean ±s.d.) while the second system was reduced to a pH of 7.53 ± 0.06 (pCO_2_ = 1,297 ± 143 μatm). The increase in CO_2_ concentration, and reduction in pH, occurred gradually, over the course of 5 days in order to mitigate animal stress, and was accomplished by bubbling CO_2_ into the treatment header tank. The addition of CO_2_ was controlled by a micro pH controller (Reef Fanatic, accuracy 0.01 pH, IN, USA) and the pH and temperature were recorded daily for the header tanks and each experimental tank using a portable probe (Hach HQ40d, accuracy 0.01 pH, CO, USA). Weekly water samples were taken from each header tank and four randomly selected experimental tanks (2 ambient pH and 2 reduced pH), then submitted to the Dickson laboratory at SIO for analysis of pH, total alkalinity, and density based salinity (for water chemistry data, see supplementary material, Table S1)

The experiment was run for 21 days after the experimental pH level was reached, during which shrimp were fed commercially available fish flakes (TetraMin) three times per week, with uneaten flakes removed. Shrimp were checked twice daily, with moults recorded and exuvia removed immediately. All of the removed exuvia were complete and showed no signs of animal consumption. At the end of the experiment, the body mass and carapace length of each shrimp were measured as described above.

### Cuticle thickness and mineral content

Shrimp were anesthetized using hypothermia by brief placement in a −20 oC freezer until showing no visible movement and then euthanized by quickly cutting off the abdomen with a razor blade. Each shrimp was briefly examined for moult stage under the microscope during dissection by checking for apolysis at the base of the uropod setae. None of the shrimp showed signs of apolysis, and thus entering the next premoult phase. The carapace was then removed from each shrimp and the underlying epidermis carefully scraped off with a small paint brush. Carapace cuticle was used because of its large size, protective function, and importance in camouflage. To examine the cross-section of the carapace cuticle, the carapace was freeze-fractured with liquid nitrogen to obtain a clean break. Cuticle specimens were then placed in a critical point dryer, secured to a double 90° SEM mount revealing the cross-section, sputter-coated with iridium, and examined using a scanning electron microscope (SEM) (Phillips XL30 ESEM, FEI Co., Hillsboro, OR, USA) equipped with an energy dispersive x-ray analysis system (EDX) (Oxford Instruments, UK).

SEM imaging was done at 10 kV acceleration voltage. The cross-section of the cuticle was examined in three separate locations on each carapace sample. At each location, the total thickness of the cuticle (i.e., epicuticle, exocuticle, endocuticle, and membranous layer), along with the thickness of each individual layer, when possible, was measured using SEM software. EDX was used to both quantify and map the distribution of elements in the cuticle cross-section. For each of these analyses, only samples with clean breaks and relatively smooth surfaces were used. The cuticle image was magnified so that it filled the screen and the spectra and element maps were all collected at a 20 kV acceleration voltage and a minimum of 5,000 counts per second. A semi-quantitative analysis of all elements in the cuticle cross-section was made using EDX spectra from up to three cuticle locations on each sample. Six elements were consistently detected in samples (C, O, Na, Mg, Cl, Ca), sometimes combined with Br, S, and Al. The amount of calcium and magnesium in each sample was calculated as the weight percent (wt%) and atomic percent (at%) relative to all detected elements, excluding the Ir coating. In addition, EDX element maps were taken from one location on each sample and used to visualize the distribution and density of calcium and magnesium in the cuticle cross-section.

### Statistical analysis

The means of percent growth (carapace length), cuticle thickness, layer thicknesses, weight percent calcium, weight percent magnesium, and the Ca:Mg ratios were compared across animal size and between ambient and reduced pH conditions. All data were tested for normality using the Shapiro-Wilk test and for homogeneity of variances using Bartlett’s test. Between treatment comparisons for normally distributed data were made using a Welch two sample t-test, otherwise a Wilcoxon test was used. Linear regression was used to compare variables across animal size and ANCOVA was used to test for differences between treatments. Percent growth was log-transformed prior to linear regression. Descriptive statistics (N values and mean ±s.d.) and statistical tests were performed in R version 3.0.2.

## Results

### Moulting and growth

**Figure 1:**
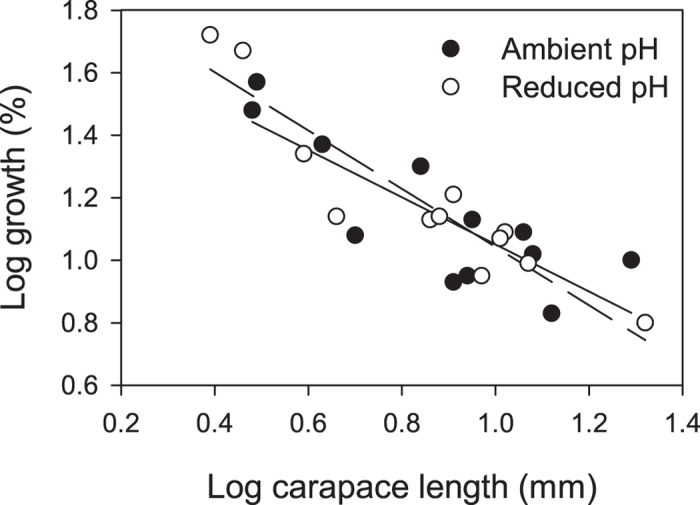
Percent growth in carapace length for shrimp in ambient and reduced pH. Slopes for log-transformed data did not differ between shrimp in ambient and reduced pH conditions.

All animals survived the 21 day experiment, during which 10 out of 12 animals from each group moulted. Three individuals moulted twice, two from ambient and one from reduced pH conditions. Three animals in reduced pH conditions that moulted during the last four days were included in growth analyses, but excluded from cuticle analyses to ensure that the new cuticle had sufficient time to form; the California brown shrimp, *Panaeus californiensis*, continues secreting matrix up to 96 hours postmoult^38^. The mean percent growth in carapace length for shrimp in reduced pH conditions was 8.58% (±15.28%, *N* = 12), which was not significantly different from that of shrimp in ambient pH, 6.13% (±9.65%, *N* = 12) (Wilcoxon signed-rank test: *P* = 0.63). Percent growth in carapace length decreased with increased body size for shrimp in both ambient [least-square regression (LSR): slope = –0.75, d.f. = 10, *R*^*2*^ = 0.68, F = 21.6, *N* = 12, *P* < 0.001] and reduced pH (LSR: slope = –0.93, d.f. = 10, *R*^*2*^ = 0.86, F = 60.9, *N* = 12, *P* < 0.0001). The regression lines did not differ between the pH conditions (ANCOVA: F = 0.1, *N* = 12, *P* = 0.94) (Fig. 1).

### Cuticle structure and calcification

**Figure 2:**
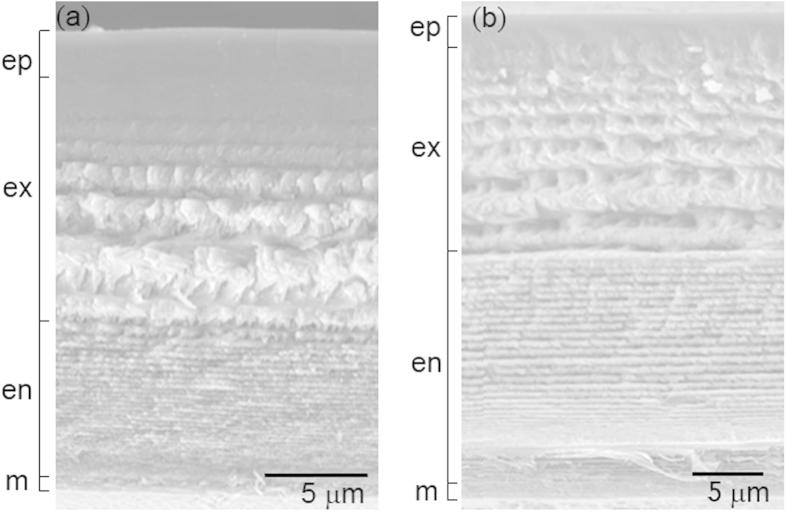
Scanning electron micrographs of cuticle from shrimp in (**a**) ambient and (**b**) reduced pH. Cuticle layers are noted on left and approximate thickness. There were no visible disruptions to cuticle structure when exposed to reduced pH. ep = epicuticle, ex = exocuticle, en = endocuticle, m = membranous layer.

**Figure 3:**
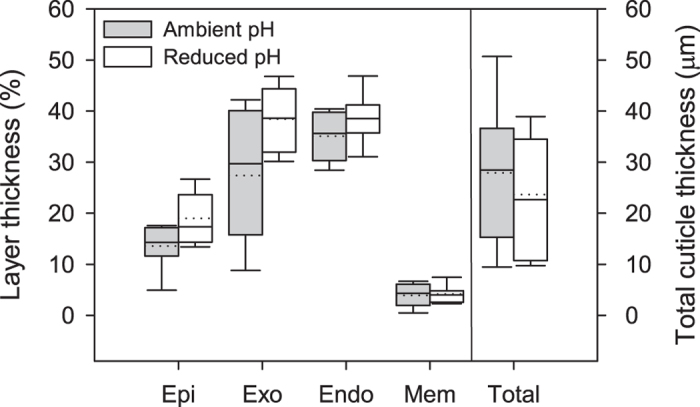
Cuticle thickness of shrimp in ambient and reduced pH. Cuticle thickness was taken as the total thickness of all layers, and did not differ between shrimp in ambient and reduced pH conditions. Total cuticle thickness in μm. Individual layer thickness is represented as percent of total cuticle thickness and no layers differed between pH treatments. Box boundaries = 25^th^ and 75^th^ percentile, error bars = 10^th^ and 90^th^ percentile, solid line = median, dotted line = mean.

SEM imaging revealed no visible differences in structure across the layers of the carapace cuticle in shrimp from ambient and reduced pH conditions (Fig. 2a,b). All cuticle layers were distinguishable, with relatively thin epicuticle and membranous layers and thicker exocuticle and endocuticle layers. The exocuticle consistently exhibited an inward gradient of increasing lamellae thickness, whereas the lamellae of the endocuticle were uniform and much thinner, with a greater stacking density. The mean total cuticle thickness was not significantly different between shrimp in ambient (27.92 μm ±13.32) and reduced pH (23.66 μm ±11.24) (t-test: *N* = 12, 9, *P* = 0.39). There was also no difference in the mean thickness of individual cuticle layers as a percent of total cuticle thickness between animals kept at ambient pH (epi = 16.97% ±8.51, exo = 27.58% ±13.07, endo = 31.35% ±12.16, mem = 3.51% ±2.07) and those kept at reduced pH (epi = 17.43%±5.87, exo = 37.75% ±15.99, endo = 36.09% ±10.05, mem = 4.14% ±2.26) (ANCOVA: *N* = 11,8, *P* = 0.38) (Fig. 3).

**Figure 4:**
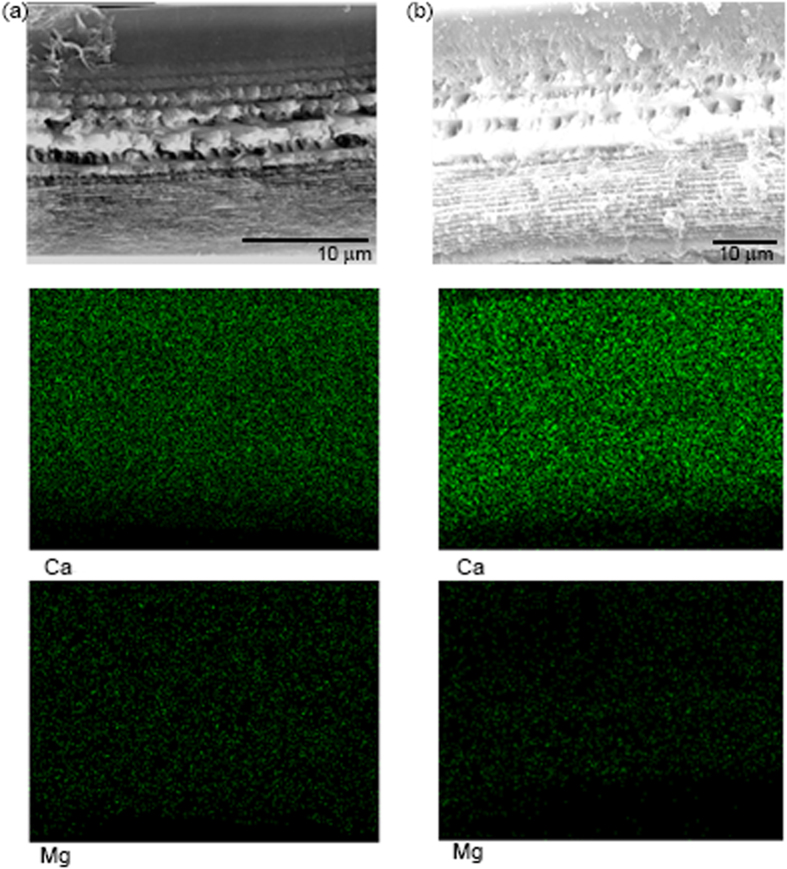
Mineral maps of Ca and Mg in shrimp cuticle from (**a**) ambient pH and (**b**) reduced pH. Ca density is uniform across the cuticle but greater in reduced pH. Mg density and distribution is similar for both pH treatments.

**Figure 5:**
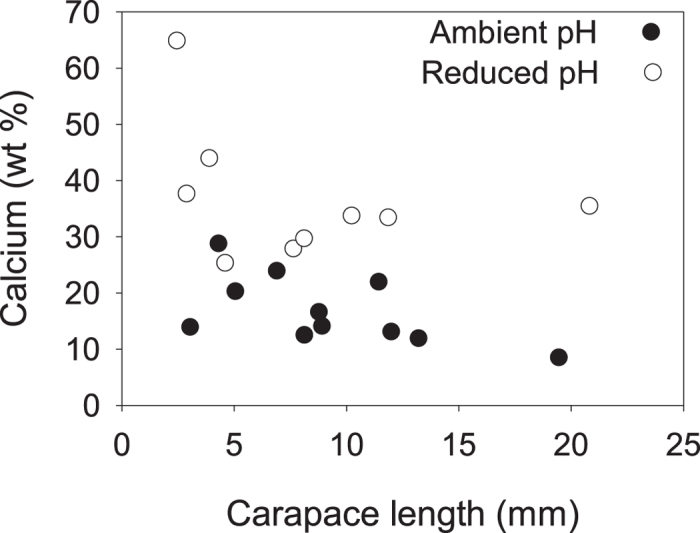
Scaling of calcium content. The percent calcium across the cuticle did not vary with animal size, but was greater in animals from reduced pH.

**Figure 6:**
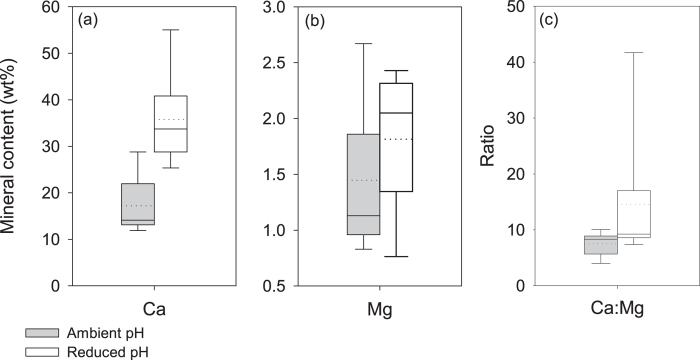
Cuticle mineral content from shrimp in ambient and reduced pH conditions. (**a**) Percent Ca was significantly greater in reduced pH. (**b**) Percent Mg was minimal and the same across pH treatments. (**c**) The Ca:Mg ratio was significantly greater in reduced pH conditions. Box boundaries = 25^th^ and 75^th^ percentile, error bars = 10^th^ and 90^th^ percentile, solid line = median, dotted line = mean.

EDX elemental mapping shows that calcium was distributed uniformly across the cuticle in shrimp from ambient pH and reduced pH, but the density (viz. calcium concentration) was greater in the cuticle of shrimp in reduced pH (Fig. 4). Magnesium was also uniformly distributed, but the density was similar in shrimp from both pH conditions (Fig. 4). The weight percent calcium was constant across animal size, with animals in reduced pH having higher levels of calcium (Fig. 5). Shrimp maintained in reduced pH had 35.8 wt% ±9.1 calcium across the cuticle, which was significantly more and approximately two times the amount of calcium in ambient pH shrimp (17.2 wt% ±6.1; t-test: *N* = 11,9 *P* < 0.001) (Fig. 6a). Percent magnesium remained low and consistent for both pH conditions (ambient: 1.45 wt% ±0.67; reduced: 1.82 wt% ±0.59; T-test: *N* = 11,9 *P* = 0.27) (Fig. 6b), thus, the ratio of Ca:Mg (at%) increased for shrimp in reduced pH due only to the increase in calcium (Fig. 6c).

### Shrimp biophotonics

**Figure 7:**
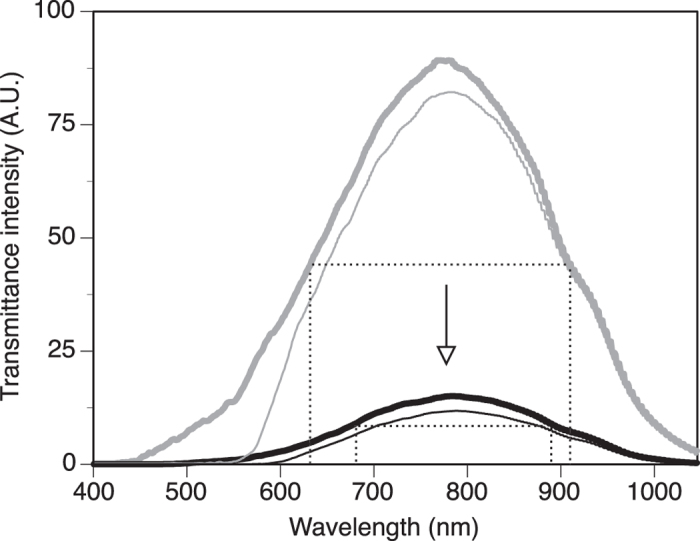
Representative transmittance spectra for control (grey) and low-pH treated (black) specimens, from the body (thick line) as well as small features associated with the cuticle (hair, holes, ridges; thin line). The dotted lines identify the spectral range at half of the maximum peak intensity for the body transmittance of control (grey) and low-pH treated (black) specimens. The arrow shows the overall trend of change in transmittance between the control and low-pH treated specimen.

Shrimp transparency was affected by reduced pH, both in terms of the spectral profile as well as the level (intensity) of transparency (Fig. 7). Control samples (grey lines in Fig. 7, mapping two different areas) showed body transparency peaking from ≈630-910 nm (measured at half of the maximum peak intensity; dotted lines) while at lower pH, this range of transparency was more narrow, extending from 680-885 nm only, thus reducing down the spectral range passing through the shrimp (black lines in Fig. 7). In other words, these data indicate that the overall shrimp changed chemical/structural make-up, resulting in narrowed spectral transmittance. In addition to the spectral range of transparency being narrowed under lower pH, the transparency overall was decreased in intensity by a factor of 5x-7x (black lines of the spectrum have lower intensity, thus showing less transparency). This indicated a decrease in transparency (or increase in opacity) to all wavelengths, especially in the blue ranging from 450-600 nm.

**Figure 8:**
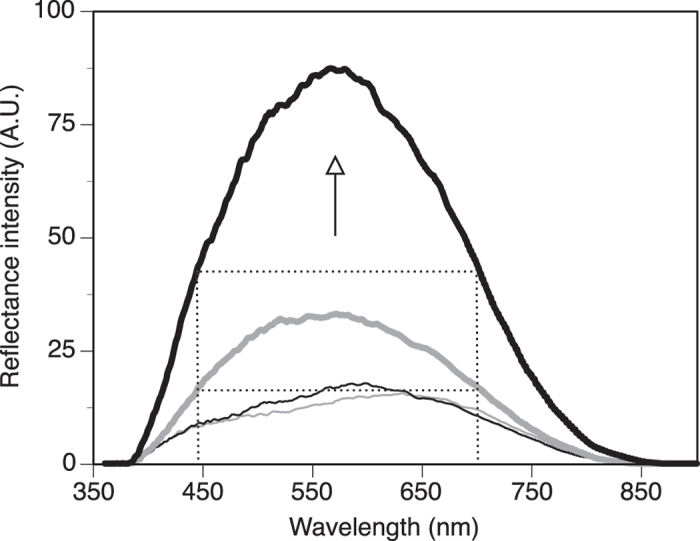
Representative reflectance spectra (in specular mode) for control (grey) and low-pH treated (black) specimens, from the body (thick line) as well as small features associated with the cuticle (hair, holes, ridges; thin line). The dotted lines identify the spectral range at half of the maximum peak intensity for the body reflectance of control (grey) and low-pH treated (black) specimens. The arrow shows the overall trend of change in reflectance between the control and low-pH treated specimen.

In contrast, shrimp color in specular reflectance showed limited changes with pH treatment, although the color of some of the colored areas could be up to 3x-5x more intense (Fig. 8). Control samples showed shrimp with a dominant color at about 525-615 nm, with some features having green spectra (at around 500 nm) but also reddish spectra (at around 650 nm; Fig. 8). At lower pH, these color spectra remained the same, and associated with spotted features. Thus, overall the color wavelength did not show significant changes with lower pH. Similarly, the intensity of the color did not change with the short-term reduced-pH treatment, although being 3x-5x brighter for specific spotted areas (compare the grey and black spectra in Fig. 8).

## Discussion

Crustaceans are generally thought to be tolerant of fairly broad changes in ocean carbon chemistry, such as those associated with ocean acidification or upwelling events, potentially because of their pH regulation abilities, less soluble form of CaCO_3_, and biological control of the calcification process^14^,^15^,^16^,^17^. It is evident, however, that crustaceans, like other marine organisms, show a variety of responses to both short-term and long-term changes in ocean carbon chemistry, including increased calcification rates^26^. This study on the caridean shrimp, *L. californica*, shows that moulting and growth are not affected in the short-term, but that there are significant changes in exoskeleton calcification and shrimp transparency within just three weeks of exposure to reduced pH.

The crustacean moulting process is highly susceptible to environmental factors, particularly temperature and food availability, which affect growth by altering moult frequency and moult increment^39^,^40^. While the relationships between temperature, food, and growth are consistent, the response to CO_2_-induced pH reduction is variable. Even in closely related prawn species exposed to reduced pH, moult frequency declined in one (*Penaeus monodon*), but not the other (*P. occidentalis*)^20^. This study on *L. californica* was too short to evaluate moult frequency, which, in the cogener species *L. seticaudata*, is 14.5 to 20 day^41^. Except for an immediate increase in moult frequency, an effect wouldn’t be detectable on this short time scale. In fact, it may take months for changes in moult frequency to be detected, even in animals that moult this frequently. For example, in the shrimp *Palaemon pacificus* a decrease in moult frequency occurred after 10-12 weeks of exposure to high pCO_2_ levels (1,900 ppmv), whereas an increase in moult frequency wasn’t observed until 21 weeks for animals exposed to intermediate pCO_2_ levels (1,000 ppmv)^24^. Most individuals (83% from each treatment) moulted successfully during this short study, suggesting that the reduced pH had no impact on the ability to moult.

The second component of growth, moult increment, was unaffected by reduced pH in our experiment, averaging less than 10% for shrimp in both pH conditions. This moult increment is comparable to that of other crustacean species^42^. Furthermore, *L. californica* showed a typical, logarithmic growth curve, with larger moult increments in smaller individuals. These growth curves also did not differ between pH conditions. Potential impacts on moult increment could be evident as early as the first moult, as was observed in juvenile tanner crabs^22^. While a reduced moult increment was observed in the first moult of tanner crabs exposed to a pH 7.5, king crabs did not show a reduction until the third moult^22^. For *L. californica*, it was hypothesized that increased calcification would be associated with increased growth, as suggested by Ries and collaborators^21^. In that study, greater calcification rates were observed under high pCO_2_ conditions for a crab, prawn, and lobster species, which were all larger by the end of the 60 day experiment. Measurements of net calcification, however, were taken from changes in buoyant animal weight and dry exoskeleton weight, thereby giving no indication if the increased weight reflected larger body size or simply a heavier, more mineralized exoskeleton. Our data on *L. californica,* a species of caridean shrimp, show that there is no corresponding increase in linear body size, i.e. exoskeleton dimensions, to account for increases in calcification. For *L. californica*, the early response to reduced pH does not manifest in changes in linear growth.

When exposed to reduced pH, the calcification of *L. californica* increased significantly, consistent with findings of previous studies on other crustacean species^18^,^19^,^20^,^21^,^43^. The mean percent calcium across the thickness of the cuticle increased from 18 wt% under ambient pH to 36 wt% in shrimp exposed to reduced pH, and was constant across animal size, which is what was previously observed in crayfish^44^. This percentage of calcium measured for shrimp in ambient pH is similar to values obtained for other shrimps, such as *Panaeus californiensis* (12.5%)^34^ and *Metapenaeus* sp. (19%)^45^, but less than that of a more heavily calcified crustacean such as the blue crab, *Callinectes sapidus* (29%)^46^ . The magnitude of change in percent calcium observed in *L. californica* (50%), is much larger though than that found in prawn carapace, which amounted to less than a 5% increase for a comparable change in pH^20^. This difference may be attributed to species specific responses, variation in experimental conditions, or the different methods used to analyze calcium content. Atomic mass spectrophotometry quantifies elements per unit volume of material whereas energy dispersive x-ray analysis (EDX) calculates relative atomic and weight percent of elements across a 2-dimensional surface. EDX has the benefit of mapping elements across a surface, providing useful information about the distribution of elements. In *L. californica*, both calcium and magnesium were distributed uniformly across the calcified layers, suggesting that the increased calcium observed under reduced pH conditions was not confined to one particular layer. Both EDX spectra and mapping functions revealed similar conclusions about the relative amount of calcium in shrimp carapace cuticle. EDX analysis, combined with scanning electron microscopy imaging, has been successfully used to determine detailed patterns of postmoult calcification in blue crabs, *Callinectes sapidus,*^33^ and the mineral patterns of the smashing appendage of the mantis shrimp^47^. Here it successfully showed consistent and significant increases in the relative amount of calcium in shrimp exposed to reduced pH, while also revealing the uniform distribution of calcium.

Magnesium is commonly found in crustacean cuticles in small amounts, and may be used as a substitute for calcium^48^. The solubility of this magnesian-calcite increases with Mg content and is greater than that of pure calcite^49^. In barnacles exposed to ocean acidification conditions, loosely bound magnesium is lost more quickly than calcium^43^. Using EDX analysis, it was determined that the carapace cuticle of *L. californica* maintains consistent, low levels of magnesium, 1.61 and 1.69 wt% in shrimp from ambient and reduced pH, respectively. These values are slightly higher than the 0.99% magnesium found in the brown shrimp, *Panaeus californiensis*^34^ and more similar to that found in the blue crab, *Callinectes sapidus* (1.7%)^46^. Like in the prawn, *P. monodon*, magnesium levels in *L. californica* were unaffected by CO_2_-induced acidification^20^. Thus, the Ca:Mg ratio increases significantly, but only due to the large increase in calcium, suggesting the presence of a less soluble form of calcite.

Interestingly, there were no observable effects on cuticle structure, nor was there a corresponding increase in cuticle thickness or relative thickness of the calcified cuticle layers of the shrimp carapace. This observation has not been described in other studies on crustaceans, primarily because net calcification rates are mostly determined through changes in exoskeleton dry weight or spectrophotometry (increased opacity measurements). For calcium content per unit cuticle thickness to increase, then mineral density must increase without a corresponding increase in matrix material. Normal increases in exoskeleton thickness involve the deposition of matrix along with calcium, so that the calcium concentration remains constant with increased size^38^. When exposed to reduced pH, however, this pattern is disrupted, with continued, disproportionate increases in calcium content. Further detailed study focusing on smaller scale ultrastructural changes of the cuticle is warranted.

Increased calcification could present a mechanical (and ecological) problem for shrimp, because when the mineral to matrix ratio changes, so do the material properties. In general, increasing the mineral to matrix ratio will make biological structures more stiff (and heavier), but also more brittle, because the matrix is important for reducing crack propagation^47^,^50^. In the raptorial appendage of the mantis shrimp, *Gonodactylus chiragra*, which produces exceptionally fast and powerful strikes against prey and opponents, the outermost impact surface is heavily mineralized, yet breaks in a brittle manner^47^. The carapace of *L. californica* primarily serves to protect the internal organs and may fracture more easily during predatory attempts. If similar changes in calcification occur in other regions of the body that experience impulsive loads, such as the abdomen during the powerful tail-flip escape response characteristic of shrimp, the risk of fracture may increase. The mechanical functioning of the crustacean exoskeleton as its composition changes during CO_2_-induced pH reduction is an important link to behavior and ecology that needs further research.

As the mineral content of the exoskeleton increases, so too does the density of the exoskeleton, which may cause discontinuities in the refractive index. This in turn can disrupt the light scattering ability of animals, the minimization of which is essential for attaining transparency^37^. Thus, transparency can be reduced as a result of increased cuticle mineralization. Indeed, we found a 5 to 7-fold decrease in transparency in shrimp exposed to ocean acidification conditions. This difference in transparency, however, cannot be attributed solely to the exoskeleton, because transmittance was measured through the entire body, the tissues of which can also contribute to the optical properties. A change of this magnitude is potentially enough to increase the distance that shrimp are detectable to visual predators, thereby raising the risk of increased predation. Large decreases in transparency of shrimp have also been observed with changes in other environmental variables; in the estuarine grass shrimp, *Palaemonetes pugio*, transparency decreased with increased temperature and salinity^51^. In conjunction with our findings that reduced pH decreases transparency, it is possible that the impact on crypsis may be compounded with the expected, concurrent increases in water temperature and salinity due to global warming.

Though transparency decreased, there was no overall change in the reflectance spectra and intensity of the red stripes characteristic of *L. californica*. Chromatophores in these shrimp occur in the hypodermis underlying the exoskeleton, and it was therefore hypothesized that changes in cuticle mineralization and transparency would affect color intensity. The chromatophores, which give these shrimp their color, can be adapted at either quick physiological or slow morphological time scales^52^. The ability to modify chromatophores allows shrimp to camouflage against different backgrounds^53^. It may be that the shrimp in this study modify their chromatophores in response to reduced pH, thereby maintaining color intensity or even increasing it in limited areas. This would suggest a functional importance of the red stripe intensity. It is assumed that disruptive coloration is associated with critical visual functions of recognition or anti-predation^35^,^36^. However, the adaptive significance of the red stripes for *Lysmata* spp. is unclear. *L. californica* is an opportunistic cleaner^54^,^55^, and does not possess the bright colors or spots nor the waving behavior characteristic of obligate cleaner shrimp species that use these mechanisms to attract customers^56^,^57^. Furthermore, mates are typically found through chemical signaling, rather than coloration^52^. It is possible that the red stripes help camouflage through disruptive coloration against the background of their rocky habitat.

In conclusion, we found that a species of caridean shrimp responds relatively quickly to decreases in water pH, doubling the amount of calcium in the carapace exoskeleton. Shrimp transparency decreased up to 7-fold, but the red coloration remains unaffected. Increased mineralization and reduced transparency in response to reduced pH could be a general consequence for many transparent, calcified crustaceans that inhabit upwelling regions or, more generally, inhabit areas that will be impacted with forecasted ocean acidification. More studies are needed to determine the mechanical and ecological consequences of animals whose calcified parts are impacted by ocean acidification and other environmental stressors.

## Additional Information

**How to cite this article**: Taylor, J. R. A. *et al.* Effects of CO^2^-induced pH reduction on the exoskeleton structure and biophotonic properties of the shrimp *Lysmata californica. Sci. Rep.* **5**, 10608; doi: 10.1038/srep10608 (2015).

## Supplementary Material

Supporting Information
