# Supplementary material for: Effects of CO2-induced pH reduction on the exoskeleton structure and biophotonic properties of the shrimp Lysmata californica
Source: Sci Rep. 2015 Jun 1;5:10608. doi: 10.1038/srep10608 (PMC4450593; doi:10.1038/srep10608)
Supplement: Supporting Information [file srep10608-s1.doc]

**Effects of CO_2_-induced pH reduction on the exoskeleton structure and biophotonic properties of the shrimp *Lysmata californica***

Taylor, J. R. A., Gilleard, J. M., Allen, M. G. & Deheyn, D. D.

**Supplementary Information**

Methods

*Animals:* The red rock shrimp, *Lysmata californica* (Caridea: Hippolytidae), was selected because it is locally abundant and easy to collect and maintain in aquaria, making it a good experimental model. *L. californica* shares characteristics of many other common shrimp, such as occupying shallow water benthic habitats, possessing habitat-specific coloration patterns, and participating in symbiotic relationships with other animals*. L. californica* inhabit rocks and crevices in low intertidal and subtidal zones from California to Galapagos Islands^1^, in areas that regularly experience upwelling. They provide cleaning services to fish by removing parasites, and are often found in association with moray eels. Their bodies are semi-translucent with bright red bands (Fig. S1).


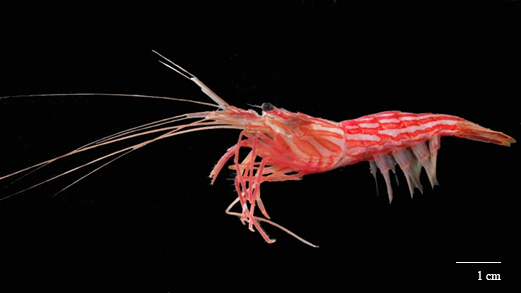


Figure S1. The red rock shrimp, *Lysmata californica*. Photograph courtesy of D. D. Deheyn and M. C. Allen.

*Water chemistry:* Water pH, salinity, and total alkalinity were measured for both ambient pH and reduced pH aquaria by the Dickson laboratory at SIO. Bicarbonate (HCO_3_^-^), carbonate (CO_3_^2-^), pCO_2_, and the saturation states (Ω) of calcite and aragonite were then calculated from these parameters using CO2calc [Robbins et al., 2010] with constants from Merhbach et al.^2^ refit by Dickson and Millero^3^ (Table S1).

**Table S1.** Means and standard deviations of water parameters over the course of the experiment. Measured parameters are noted with an asterisk. All other parameters were calculated using CO2calc.

|  | pH^*^ | Salinity^*^ | Total^*^ alkalinity  (µmol kg^-1^) | HCO_3_  (µmol kg^-1^) | CO_3_  (µmol kg^-1^) | pCO_2_  (µatm) | Ω Ca | Ω Ar |
| --- | --- | --- | --- | --- | --- | --- | --- | --- |
| Ambient pH | 7.99  (0.04) | 33.631  (0.019) | 2234.95  (5.07) | 1869  (8.5) | 148  (2.6) | 462  (11) | 3.57  (0.06) | 2.31  (0.04) |
| Reduced  pH | 7.53  (0.06) | 33.629  (0.024) | 2234.24  (4.45) | 2066  (16) | 68  (6.8) | 1297  (143) | 1.65  (0.16) | 1.07  (0.11) |


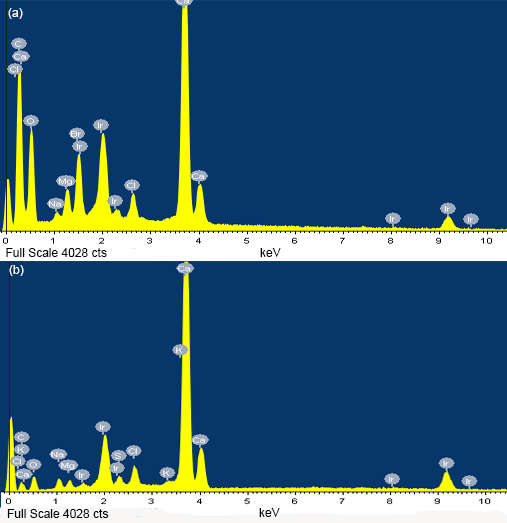


**Figure S2**. Sample EDX spectra from cuticle cross-section in shrimp from ambient pH (a) and reduced pH (b) conditions. Spectra taken at 20 keV, but images cropped to 10.5 keV. Elemental peaks were consistently detected for O, C, Na, Mg, Cl, Ca and sometimes Br, S, and P. Ir peaks are identified but were not included in quantitative analyses. In these particular sample spectra, the Ca in relation to all detected elements was 13.94 wt% in ambient pH (a) and 53.89 wt% in reduced pH (b).

**Literature cited**

1. Jensen, G. C. *Pacific Coast Crabs and Shrimps.* Monterey: Sea Challengers (1995).

2. Mehrbach, C., Culberson, C.H., Hawley, J.E. & Pytkowicz, R.M. Measurement of the apparent dissociation constants of carbonic acid in seawater at atmospheric pressure. *Limnol. Ocean*. **18,** 897-907 (1973).

3. Dickson, A. G. & Millero, F. J. A comparison of the equilibrium constants for the dissociation of carbonic acid in seawater media. *Deep Sea Res.* **34**, 1733-1743 (1987).
